# Metformin down-regulates epithelial-mesenchymal transition (EMT) in fibroblasts from burned patients

**DOI:** 10.1186/2049-3002-2-S1-P84

**Published:** 2014-05-28

**Authors:** Michael Wetzel, David Herndon, Celeste Finnerty

**Affiliations:** 1 Cell Biology Graduate Program, University of Texas Medical Branch, Galveston, TX, USA; 2 Surgery, University of Texas Medical Branch, Galveston, TX, USA; 3 Shriners Hospital for Children, University of Texas Medical Branch, Galveston, TX, USA; 4 Institute for Translational Sciences and Sealy Center for Molecular Medicine, University of Texas Medical Branch, Galveston, TX, USA

## Background

Burn injury is a significant problem that affects approximately half a million people in the U.S. annually. One of the major complications emerging from burn injury is hyperglycemia, which can last for weeks following the initial trauma. Current therapeutic methods include tight euglycemic control and insulin treatment, which are associated with increases in hypoglycemia and mortality. High glucose levels decrease wound healing, while decreased glucose with insulin improves it. Alternatives to insulin therapy include biguanide drugs such as metformin. Metformin downregulates gluconeogenesis, and reduces blood glucose levels by activating GLUT4 in muscle cells. Recent work has also shown that metformin decreases keratinocyte proliferation *in vitro*. As the burn wounds in our patients typically yield severe hypertrophic scars, which result from a hyperproliferation of fibroblasts, we have studied the effect of metformin on wound healing, proliferation, and epithelial-mesenchymal transition (EMT). We hypothesize that biguanides will decrease EMT, thereby promoting a less fibrogenic state called MET, in fibroblasts derived from normal skin and burn skin and scar, resulting in decreased scarring. Here we examine the effects of metformin on cellular proliferation and EMT.

## Materials and methods

Fibroblasts were isolated from skin and burn scar from pediatric burn patients, and non-burned fibroblasts purchased from ATCC. Cells were treated with or without 5 mM metformin. Proliferation following metformin exposure was quantitated with the xCelligence™ (Acea Biosciences) and EMT gene expression determined using quantitative real time PCR.

## Results

We found that metformin down regulates genes related to fibrosis and EMT. Quantitation of the expression of TGF-β, collagen, and SMAD2 and 3 showed decreases in EMT and fibrosis genes following metformin treatment. Furthermore, proliferation of the fibroblasts was decreased with metformin administration.

## Conclusions

These results indicate that metformin alters the fibroblast phenotype, reducing the proliferative potential of these cells, and decreasing EMT and fibrosis. This may indicate that metformin is suitable for decreasing the hyperproliferative state that leads to pathological scarring. Further studies will allow a better understanding of the effects of metformin on fibroblasts leading to new therapeutic options for scar management in burn patients.

